# Moderate hypoxia influences excitability and blocks dendrotoxin sensitive K^+ ^currents in rat primary sensory neurones

**DOI:** 10.1186/1744-8069-2-12

**Published:** 2006-03-31

**Authors:** Marco Gruss, Giovanni Ettorre, Annette Jana Stehr, Michael Henrich, Gunter Hempelmann, Andreas Scholz

**Affiliations:** 1 Physiologisches Institut, Justus-Liebig-Universität, 35385 Giessen, Germany; 2 Abteilung Anaesthesiologie, Intensivmedizin, Schmerztherapie, Universitätsklinikum Gießen und Marburg, Standort Gießen, 35385 Giessen, Germany; 3 Zentrum für Anaesthesiologie, Rettungs- und Intensivmedizin, Robert-Koch-Str.40, 37075 Göttingen, Germany; 4 University Laboratory of Physiology, Parks Road, Oxford OX1 3PT, UK

## Abstract

Hypoxia alters neuronal function and can lead to neuronal injury or death especially in the central nervous system. But little is known about the effects of hypoxia in neurones of the peripheral nervous system (PNS), which survive longer hypoxic periods. Additionally, people have experienced unpleasant sensations during ischemia which are dedicated to changes in conduction properties or changes in excitability in the PNS. However, the underlying ionic conductances in dorsal root ganglion (DRG) neurones have not been investigated in detail.

Therefore we investigated the influence of moderate hypoxia (27.0 ± 1.5 mmHg) on action potentials, excitability and ionic conductances of small neurones in a slice preparation of DRGs of young rats. The neurones responded within a few minutes non-uniformly to moderate hypoxia: changes of excitability could be assigned to decreased outward currents in most of the neurones (77%) whereas a smaller group (23%) displayed increased outward currents in Ringer solution. We were able to attribute most of the reduction in outward-current to a voltage-gated K^+ ^current which activated at potentials positive to -50 mV and was sensitive to 50 nM α-dendrotoxin (DTX). Other toxins that inhibit subtypes of voltage gated K^+ ^channels, such as margatoxin (MgTX), dendrotoxin-K (DTX-K), r-tityustoxin Kα (TsTX-K) and r-agitoxin (AgTX-2) failed to prevent the hypoxia induced reduction. Therefore we could not assign the hypoxia sensitive K^+ ^current to one homomeric K_V _channel type in sensory neurones. Functionally this K^+ ^current blockade might underlie the increased action potential (AP) duration in these neurones. Altogether these results, might explain the functional impairment of peripheral neurones under moderate hypoxia.

## Background

In the last years the knowledge about the effects of hypoxia on different organ systems showed a remarkable increase. Most efforts were made to understand the actions of hypoxia in the central nervous system, in the regulation of blood flow, in the sensing of oxygen levels in chemoreceptors of the carotid body and the neuroepithelial bodies of the lung (see [1-3] for reviews).

Besides the manifold effects of chronic hypoxia on gene-regulation and gene-expression, mechanisms leading to long-term changes and adaptation of cell metabolism [2], it has been established that acute hypoxia modulates the activity of a wide range of different ion channels. Modulation of Ca^2+ ^channels [4,5], Na^+ ^channels [6,7] and especially K^+ ^channels [8-12] have been described to be oxygen sensitive. Surprisingly, a great variety of channel responses to hypoxia has been reported depending not only on the type of channel but also on the organic system or the expression system used [2,13]. However, the molecular interaction between ion channels and the O_2 _sensor is still not clarified in its details. It has been suggested that the O_2 _sensor is closely associated with ion channels, namely K^+ ^channels, either via the α-subunit or as part of an auxiliary subunit because some channels retained their oxygen-sensitivity when studied in excised patches [14,15]. As several of the hypoxia-induced effects on ion channels can be mimicked by reducing agents it has been concluded that hypoxia influences the balance of cellular redox couples, and this modifies thiol groups and changes channel properties [16,17]. Due to the great variability in channel regulation depending on the cellular environment and the experimental conditions it seems rather likely that regulation of ion channels is modulated by interaction between an oxygen-sensing mechanism and the pore-forming channel subunit.

Among mammalian cells especially neuronal cells are known to be highly oxygen-sensitive. Whereas the effects of hypoxia on neurones of the central nervous system have been explored in broad detail during the last years [2,15,18,19], little information is available about the effect of hypoxia on the peripheral nervous system.

There is evidence that reduced blood flow leading to a reduced oxygen supply is involved in some pathophysiological reactions in the peripheral nervous system. For example, the development of neuropathic pain and radiculopathic symptoms during mechanical compression indicate that neurones in DRGs and dorsal roots react with an increased sensitivity to direct mechanical compression and hypoxia [20,21]. Interestingly, in that study the neurones in DRGs were even more sensitive to hypoxia than dorsal roots.

Other electrophysiological experiments indicate that dorsal root nerves are susceptible to hypoxic conditions especially when combined with hyperglycaemia [22]. However, hypoxia has not been proven as a contributing factor for the underlying mechanism of painful diabetic neuropathy on the peripheral nerve [23]. For healthy subjects it is known that even brief episodes of ischemia can lead to paraesthesia but not to muscle twitching due to an increase in refractoriness and an increase in action potential latency [24]. Beside the changes in pH and elevated K^+ ^during ischemia the reduced oxygen level is discussed to be an additional factor which influences the excitability and conduction properties.

For DRG neurones, Lukyanetz et al. (2003) showed that hypoxia increases the intracellular Ca^2+ ^level by activation of high-voltage gated Ca^2+ ^channels. This hypoxia-induced Ca^2+ ^increase might activate other Ca^2+ ^depending mechanisms as described for the activation of endothelial nitric oxide synthase in the presence of extracellular Ca^2+ ^[25].

As little is known about functional effects of moderate hypoxia on DRG neurones our study was performed to investigate the acute effects of moderate hypoxia on small DRG neurones which are associated with C-type and A-δ fibres [26].

A preliminary report of this data was presented elsewhere [27].

## Methods

### Preparation

**Figure 1:**
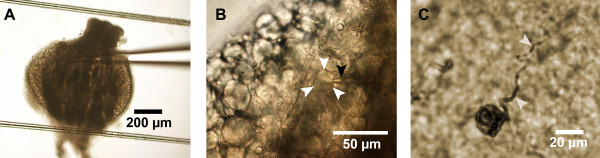
**Slice preparation of rat dorsal root ganglion**. **A) **Overview of a 150 μm slice of a DRG embedded in agar at low magnification (4×); more details about the preparation see in Methods. The two lines are strings from dental floss of the grid, holding down the slice in the experimental chamber. **B) **The same slice at higher magnification (40×) with patch pipette (black arrow) close to the neurone (white arrows). **C) **Fixation of the slice with formaldehyde (4%) after filling the neurone during whole cell recording with biocytin (0.2%) included in the patch pipette solution. Note the native axon of the sensory neurone (white arrows). Staining was visualized after incubating with the ABC-method (Avidin Biotinylated enzyme Complex).

Experiments were performed on small sized neurones (19.8 ± 0.3 μm in diameter) in thin slices of dorsal root ganglia with a thickness of about 100–150 μm of DRGs from young rats (Wistar, 2–9 d) of both sexes. The slices were prepared in ice-cold Ringer's solution without the use of enzymes and kept according to descriptions given elsewhere [28-30]. Briefly, after the rats had been decapitated (according to the German guidelines of animal care) two to four DRGs (Th10 – L5) were taken from the opened spinal column and embedded in liquid 2% (w/v) agar in Ringer's solution (40°C). After cooling in ice cold salt solution we cut small block of agar each containing one DRG. These blocks were glued to a glass plate and cut with a vibroslicer (FTB-Meier, Bensheim, Germany) at 2–6°C in ice-sludge made by Ringer's solution. Throughout the preparation the Ringer's solution was bubbled with carbogen gas (95% O_2_, 5%CO_2_). The slices were incubated in warm (34–35°C) Ringer's solution for 30 min after cutting and subsequently stored at room temperature for up to 12h. Throughout the experiments the Ringer's solution was bubbled with a mixture of 21% O_2_, 5%CO_2 _and N_2 _as balance unless otherwise stated. The present studies were restricted to neurones from the surface of the slice, because DRG neurones in the depth of the slice cannot be cleaned as described for brain slices and spinal cord [31,32]. We focused on small diameter neurones because they are mainly connected to pain-sensing nerve fibres and middle- and large-sized neurones cannot be patched because they are covered by a layer of Schwann cells [29]. Cells were visualized throughout the patch-clamp experiments by a camera and cell size was measured with a calibrated scale on a monitor at the beginning of the experiment (Fig. 1).

### Solutions

All values of concentrations are given in mM.

External solutions: **Ringer's**: NaCl 115, KCl 5.6, CaCl_2 _2.2, MgCl_2 _1, glucose 11, NaH_2_PO_4 _1, NaHCO_3 _25, final Na^+ ^141. **Choline chloride**: KCl 5.6, CaCl_2 _2.2, MgCl_2 _1, glucose 11, NaH_2_PO_4 _1, NaHCO_3 _25, choline chloride 115. **Sodium free choline chloride**: KCl 5.6, CaCl_2 _2.2, MgCl_2 _1, glucose 11, KH_2_PO_4 _1, KHCO_3 _25, choline chloride 115. The external solutions were adjusted to pH 7.4 by bubbling with a gas mixture containing about 5% CO_2_. Internal solutions: **High-K_i_EGTA**: NaCl 5, KCl 144.4, MgCl_2 _1, ethylene glycol-bis(β-aminoethyl ether)N,N,N',N',-tetraacetic acid (EGTA) 3, N-[2-hydroxyethyl]-piperazine-N'[2-ethanesulfonic acid] (HEPES) 10, adjusted to pH 7.3 with KOH, final K^+ ^155. **Potassium fluoride**: KF 100, KCl 30, Na_2_-ATP 2, HEPES 10, adjusted to pH 7.3 with KOH, final K^+ ^140.

The hypoxic solutions were bubbled either with 5% O_2 _(5%CO_2 _and N_2 _as balance) or in some experiments with oxygen-free gas (95% N_2_, 5% CO_2_). The following peptide toxins were purchased from Alomone (Israel) and diluted from stock solutions before the experiments: Margatoxin (MgTX), 1 μM in a buffer containing 100 mM NaCl, 10 mM 2-amino-2-(hydroxymethyl)-1,3-propanediol (TRIS), 1 mM ethylenediaminetetraacetate (EDTA), pH 7.5), Dendrotoxin-K (DTX-K, 10 μM in H_2_O), r-Tityustoxin Kα (TsTX-K, 1 μM in H_2_O) and r-Agitoxin 2 (AgTX-2, 1 μM in H_2_O). To avoid non-specific binding of the peptide toxins, 0.01% or 0.05% (w/v) bovine serum albumin (BSA, Sigma) was added to test solutions containing peptide toxins. Tetraethylammonium chloride (TEA-Cl) was purchased from Merck (Germany), α-Dendrotoxin (DTX, 0.14 mM in H_2_O) was a generous gift of Dr. F. Dreyer, University of Giessen. All other chemical substances were purchased from Sigma.

Experimental solutions were perfused into the main chamber using a gravity-driven perfusion system and a six-way teflon pinch valve. The partial pressures of oxygen (pO_2_) and carbon dioxide as well as the temperature and pH-value were monitored continuously throughout most experiments by a Paratrend 7 probe that measured by fluorescence changes each 1 s (Diametrics Medical Ltd., Buckinghamshire, UK). The probe was positioned close to the main chamber in the perfusion system. Before starting the experimental series we checked the pO_2 _at the bottom of the chamber where the slice was later fixed under the grid. If the flow in the chamber is as high as in our experiments the pO_2 _at the bottom of the chamber is close to that in the inlet pipe and that we could achieve "hypoxic conditions". When switching between solutions we observed small temporary changes in pH and pCO_2 _due to the change from the flowing solution to the next solution with a small amount standing in the supply tube. These changes where in general shorter than 30 s. Current recordings were obtained after 3–4 min in the steady-state of the solution exchange when the monitored pO_2 _was and the pH was stable.

### Current and voltage recordings

Patch-clamp pipettes were fabricated from borosilicate glass tubing with internal filament (GC 150F, Harvard Apparatus, UK) using a horizontal puller (Sutter, USA) and were heat-polished to final resistances of 2.2 – 11 MΩ (median 4.0 MΩ) measured with internal solution. Recordings were obtained using an Axopatch 200A patch-clamp amplifier (Axon Instruments, USA) and data were digitized with a Digidata 1200A 12bit AD/DA converter (Axon Instruments, USA) at a sampling frequency of 2 kHz and filtered at 1 kHz with a 10-pole low-pass Bessel filter if not otherwise stated. Voltage and current steps and data acquisition were controlled online by a PC/AT computer with pCLAMP8 software (Axon Instruments, USA). Whole-cell recordings were performed as described by Hamill et al. [33], and in most experiments additional series resistance compensation (55–80%) was applied after adjustment of series resistance and compensation of whole-cell capacity. Currents evoked by voltage steps were digitally corrected off-line for leakage and transients using appropriately scaled averaged episodes of recordings which were evoked by hyperpolarizing pulses.

Voltages are expressed as transmembrane potentials (V). Resting potentials of the neurones are shown next to the current-clamp recordings and are indicated by dotted lines in the figure. Deviations in action potential duration (measured at 0 mV) as well as in current measurements at the end of the voltage step at +40 mV from control to test conditions were taken as different if larger or smaller than 3% from control value. Those cells showing an increased outward-current after toxin application were excluded from further analysis (n = 3).

Evaluations, fits and statistical analysis were performed with the programs pCLAMP (6 and 8, Axon Instruments, USA), Origin 6.0 (Microcal Software, USA) and Excel 7 (Microsoft, USA). Data are expressed as mean ± standard error of the mean (S.E.M.). Statistical significance was assessed using the paired or unpaired Student's t-test where appropiate. Experiments were performed at room temperature (21 – 23°C).

## Results

### Effects of hypoxia on action potentials and threshold

**Figure 2:**
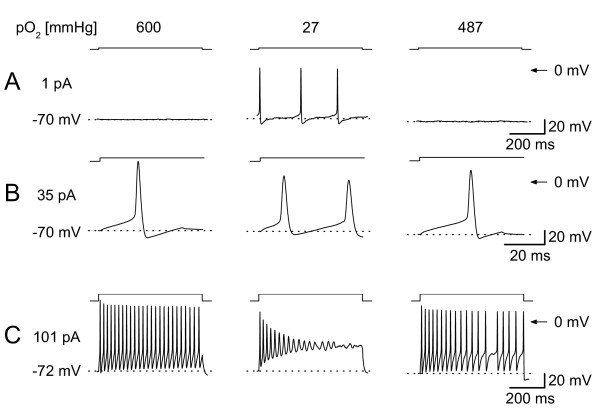
**Whole-cell recordings in the current-clamp modus**. A) A neuron which was in carbogen-bubbled control solution (pO_2 _600 mmHg, left) not spontaneous active, responded in hypoxic solution (pO_2 _27 mmHg, middle) spontaneously (1 pA). After reoxygenation the effect was reversible and the neurones did not generate an AP. B) The same neuron as shown in A. The neuron generates a second AP in the hypoxic solution compared to control (middle vs. left) but the AP-amplitude was reduced from 99 mV to 79 mV and the duration of the AP was prolonged in hypoxic solution from 2.3 ms to 2.6 ms (measured at half amplitude). C) Same neuron as shown in A and B. Trains of APs elicited in a DRG-neuron by a long lasting depolarizing current injection (750 ms, left). After reduction of pO_2 _(27 mmHg, middle) the neuron generate few APs and adapted. After reoxygenation the effect was reversible (right). 23°C; Pipette solution: High-Ki EGTA; Bath: Ringer's solution.

In patch-clamp experiments with neuronal slices (Fig. 1) most investigators use carbogen (95%O_2_, 5% CO_2_)-bubbled solutions with relatively high glucose concentrations (mostly about 10 mmol/l) to keep their cells healthy [30,34]. During some pilot experiments we tested the effect of a dramatic change in oxygen level on excitability of DRG neurones. We reduced the oxygen level in the Ringer's solution from about 600 mmHg to about 30 mmHg ("hypoxic") and measured the APs of DRG neurones. The large decrease in oxygen level resulted in a change of firing pattern as shown exemplary in figure 2. A neurone that did not show any spontaneous activity under carbogen (Fig. 2A) became spontaneously active under hypoxia and displayed an increased excitabilty when stimulated with small depolarising currents (Fig. 2B). Interestingly, when stimulated with stronger currents the neuron responded with a train of APs in carbogen-bubbled solution but showed an adapting firing behaviour in hypoxic solution in parallel we observed a decrease in outward currents. These effects were reversible after washout of hypoxic solution to carbogen-bubbled Ringer's and demonstrate the complexity of neuronal responses to hypoxia. Because only a small fraction of sensory neurones responded with repetitive firing, we focused on the effects on single action potentials.

To investigate the oxygen sensitivity of small DRG-neurones in more detail we replaced the carbogen-bubbled solution by a control bubbled with 21% O_2 _(5% CO_2_, 74% N_2_) and tested the effects of moderate hypoxia on action potentials (APs) and currents of DRG neurones.

Overall, we investigated in 137 cells the effects of moderate hypoxia on currents and actions potentials (APs) of small DRG neurones. In 98 experiments the oxygen level (pO_2_) was measured continuously throughout the experiment in control solutions (144.2 ± 0.9 mmHg), in hypoxic solutions (27.0 ± 1.5 mmHg) and in control after hypoxia (142.9 ± 1.1 mmHg; washout). In the voltage-clamp configuration we tested 127 neurones, 29 were tested with external Ringer's solution and internal High-K_i_, 33 in external choline chloride and 65 in sodium free choline chloride solution with internal potassium fluoride to investigate voltage-gated potassium currents separately.

### Different effects of moderate hypoxia on whole-cell outward currents

**Figure 3:**
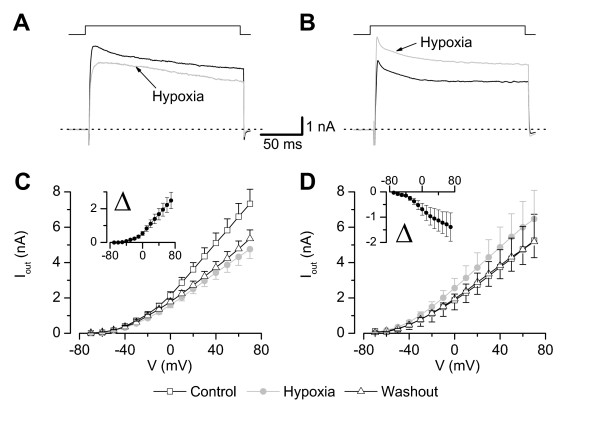
**Moderate hypoxia effects outward currents differently**. **A, B**) Current recordings (holding potential -80 mV, step to +40 mV) of neurones showing either decreased (**A**) or increased (**B**) whole-cell outward currents in hypoxic Ringer's solution. **C, D**) Current-voltage relation of neurones with reduced outward currents (**C**, 77% of neurones) or increased outward currents (**D**, 23% of neurones) in hypoxia. The insets show the I-V relation for the difference currents between control and hypoxia (**C**). Data represent mean ± S.E.M., pipette: High-K_i _EGTA, n = 23.

When we measured the effect of hypoxia (11.9 ± 4.7 mmHg) on whole-cell currents of small DRG neurones with external Ringer's solution and internal High K_i_, the majority of the neurones (22 out of 29) showed a decreased steady-state whole-cell outward current in hypoxia (3.40 ± 0.36 nA, measured at +40 mV) within tens of seconds compared to control (4.82 ± 0.43 nA, p < 0.001, Fig. 3A, C) whereas seven neurones showed an increase in outward current in hypoxia (4.87 ± 1.68 nA vs. 3.75 ± 1.35 nA in control, p < 0.05, Fig. 3B, D). The increase in outward current was reversible (p < 0.05) in washout (3.83 ± 0.83 nA, n = 6, Fig. 3D). However, the situation in the group with reduced currents was more heterogeneous; in eight neurones we observed a substantial increase in current during 3 to 6 min after washout of hypoxia whereas in other neurones the reduction was irreversible. Therefore the mean current in washout was only slightly greater than in hypoxia (3.00 ± 0.38 nA, n.s., n = 20, Fig. 3C).

### Effect of hypoxia on action potentials

**Figure 4:**
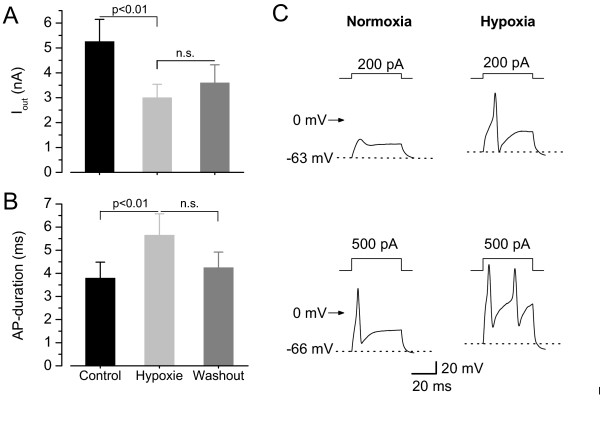
**Functional consequences of hypoxia-induced K^+ ^current block**. **A**) Outward-currents (measured at +40 mV) of neurones which showed hypoxic sensitive outward-currents (reduction by 43 %, p < 0.01) and in which we measured action potentials (n = 11). **B**) Corresponding to changes in K^+ ^currents these neurones displayed a 49% prolongation in mean AP duration in hypoxia (p < 0.01, n = 11). **C**) Hypoxia reduced the threshold to evoke an AP in some neurones. Injection of 200 pA depolarising currents could evoke an AP in hypoxia but not in normoxia and injection of 500 pA could elicit 2 APs in hypoxia compared to one in normoxia. Bath: Ringer's solution, pipette: High-K_i _EGTA.

Next, we tried to look into the functional consequences of this inhibition of K^+ ^currents by hypoxia. Neurones which showed a decreased whole-cell outward-current in hypoxic Ringer's solution (2.71 ± 0.62 nA) compared to normoxia (4.97 ± 1.18 nA; p < 0.01, n = 10, washout 2.61 ± 0.73 nA, n.s., n = 8, Fig. 4A, B) were tested in current-clamp. Currents were measured at +40 mV, which correlates to the membrane potential of the peak of an AP in many neurones. The AP duration in these neurones was prolonged (measured at 0 mV) from 3.08 ± 0.31 ms in control conditions to 5.01 ± 0.77 ms in hypoxia (p < 0.05, n = 10) and corresponding to partial recovery of the K^+ ^currents in subsequent washout (4.14 ± 0.59 ms, n = 9, n.s., Fig. 4C). The mean threshold, to evoke an AP above *V*>0 mV, did not change in hypoxia (390 ± 62 pA in control vs 360 ± 63 pA, n.s.). The resting membrane potential remained unchanged in hypoxia (-66.3 ± 2.6 mV) compared to normoxia (-68.0 ± 1.8 mV, n.s., washout 65.9 ± 2.9 mV, n.s.).

### Voltage-gated K^+ ^currents

The changes in steady-state whole-cell currents could be either due to a block or an activation of outward-currents or an increased or decreased inward-current, respectively. Several potassium channels have been shown as a possible target for hypoxia and thus we focused on the effects of moderate hypoxia on K^+ ^currents in small DRG neurones. To investigate isolated voltage-gated K^+ ^currents we used extracellular choline chloride and intracellular potassium fluoride.

**Figure 5:**
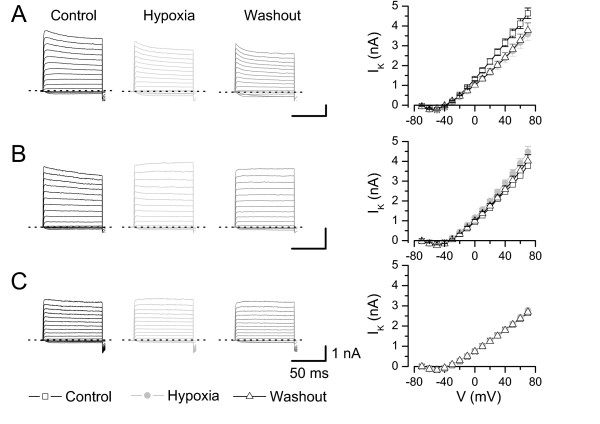
**Voltage-dependent K^+ ^currents in hypoxia**. Current recordings (holding potential -80 mV, ΔV 10 mV) in control, hypoxia and washout revealing different reactions of K^+ ^currents to hypoxia. **A**) Most neurones (63%) showed a decreased K^+ ^current during hypoxia which was irreversible in some cells (n = 17). **B**) Another group of neurones (26%, n = 7) displayed a reversible increase in K^+ ^current. **C**) Some neurones showed no (11%) or only small differences in K^+ ^currents during hypoxia (n = 3). Current-voltage relations shown right. Bath: sodium-free choline chloride, pipette: potassium fluoride.

**Figure 6:**
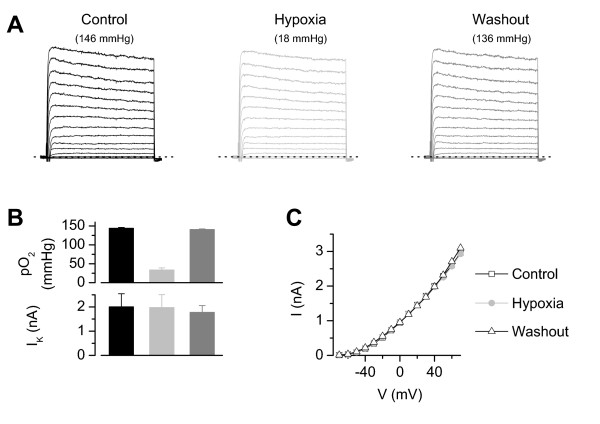
**Insensitive voltage-dependent K^+ ^currents in hypoxia**. A small group of DRG neurones (22 %) revealed no changes over time in amplitude or kinetic of voltage-gated K^+ ^currents. **A**) Whole cell recording from a neurone without changes in K^+ ^current amplitude. **B**) Summary demonstrating an unchanged K^+ ^current (lower panel, n = 7) during different pO_2_-levels (top panel). **C**) I-V relation of 7 neurones showed no changes in amplitude of K^+ ^currents over 25–35 min. Bath: choline chloride, pipette: potassium fluoride.

Again the largest group of neurones (17 out of 32) revealed a reduction of voltage-gated K^+ ^currents in hypoxia (2.37 ± 0.23 nA, at V = +40 mV, sodium free choline chloride, Fig. 5A) compared to control conditions (3.15 ± 0.19 nA, p < 0.001) which did not recover in most neurones within 3 – 6 minutes (washout 2.40 ± 0.24 nA, p > 0,05, n = 16). In contrast, eight neurones displayed a reversible increase of the K^+ ^current amplitude under hypoxia (Fig. 5B, 2.91 ± 0.15 nA compared to 2.48 ± 0.19 nA in normoxic solution, p < 0.05, washout 2.58 ± 0.17 nA, p < 0.05) whereas in three neurones the K^+ ^current remained unchanged (Fig. 5C, 1.81 ± 0.14 nA in hypoxia compared to 1.81 ± 0.16 nA in normoxia, n.s.). These experiments regarding voltage-gated K^+ ^currents support the assumption that the observed changes in whole-cell currents in Ringer's solution can be assigned mostly to effects on K^+ ^currents. Noteworthy, we observed in seven neurones (out of 32 tested) no changes in current amplitude or in the current kinetics over 25–35 min even though hypoxic solution (pO_2 _33 ± 5 mmHg) was applied meanwhile (Fig. 6). Therefore it seems unlikely that a simple "rundown" phenomenon is responsible for the decreased outward- or K^+^-currents in hypoxic solutions. In further experiments, we focused on the reduction of hypoxia-sensitive K^+ ^current during moderate hypoxia which was main group of responding neurones.

There are several different K^+ ^currents described in rat's DRG neurones [29,32,35,36] which can be partially distinguished by their physiological and pharmacological properties.

### The hypoxia-sensitive K^+ ^current is TEA-insensitive

TEA is a widely used K^+ ^channel inhibitor which is known as a relatively unspecific blocker of many voltage-gated K^+ ^channels [29,37]. Eight out of twelve neurones showed a decrease of current amplitude in hypoxic solutions from 4.12 ± 0.62 nA in choline chloride solution to 3.65 ± 0.52 nA in hypoxia (p < 0.01), whereas four neurones revealed an increase of outward current in hypoxia. In neurones with reduced current in hypoxic solution, preapplication of 5 mM TEA reduced the steady-state amplitude to 2.02 ± 0.35 nA (p < 0.001 compared to washout after hypoxia: 3.34 ± 0.48 nA). TEA at 5 mM could not abolish a further reduction to 1.79 ± 0.32 nA in TEA and hypoxia (p < 0.05).

### The hypoxic sensitive K^+ ^current is sensitive to DTX

We tested the effect of 50 nM DTX, which blocks several voltage-gated K^+ ^channels in the nanomolar range (K_V_1.1, K_V_1.2 and K_V_1.6; [37,38]).

**Figure 7:**
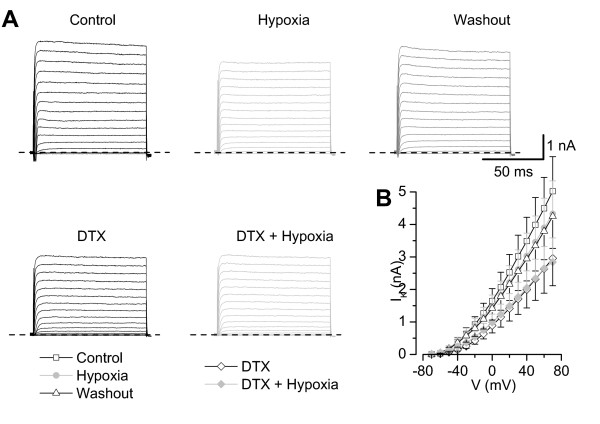
**The hypoxia-sensitive K^+ ^current is sensitive to α-DTX**. **A**) Current recordings (holding potential -80 mV, ΔV 10 mV) of a neurone showing reversibly decreased K^+ ^currents in hypoxic solution. Application of 50 nM α-DTX in normoxic solution in the same neurone prevented the reduction of K^+ ^currents in subsequently applied hypoxia. **B**) Current-voltage relation of six neurones with reduced K^+ ^currents. After application of 50 nM α-DTX the amplitude of K^+ ^currents did not further decrease in hypoxia. Bath: choline chloride, pipette: potassium fluoride.

Due to the different responses to hypoxia, we first applied the hypoxic solution to test whether the neurone displayed a reduction of K^+ ^current during hypoxia. In this case we applied subsequently DTX before a second application of hypoxic solution (Fig. 7A). With this protocol 7 out of 10 neurones showed a reduction of the mean K^+ ^current amplitude from 2.92 ± 0.73 nA in control (choline chloride) to 2.36 ± 0.69 nA in hypoxia (p < 0.01, Fig. 5B, washout 2.53 ± 0.66 nA, n.s. compared to hypoxia). Application of 50 nM DTX to the normoxic solution reduced the K^+ ^current to 1.64 ± 0.51 nA (p < 0.01) and demonstrated the presence of DTX-sensitive currents in these neurones. This preapplication of DTX completely prevented a further reduction of K^+ ^currents by hypoxia (Fig. 5, 1.60 ± 0.48 nA, n.s. compared to DTX in normoxia).

Because preapplication of DTX prevented the reduction of hypoxia-sensitive K^+^currents we compared the properties of the hypoxia-sensitive K^+ ^current with the DTX-sensitive K^+ ^current. The current to voltage (I-V) relation of the hypoxia-sensitive K^+ ^current of 17 neurones in choline chloride showed that the current activated at potentials positive to -50 mV (data not shown). In contrast, the DTX-sensitive current component described above activated at more negative potentials (i.e. positive to -70 mV). Additionally, the I-V curve of the DTX-sensitive K^+ ^current had a larger amplitude indicating that the group of DTX-sensitive K^+ ^current covers the subgroup of K^+ ^channels which were responsible for the hypoxia-sensitive K^+ ^current in small DRG neurones.

### Effects of other peptide blockers

Due to the sensitivity to 50 nM DTX, the hypoxia-sensitive current should cover K_V _1.1, K_V _1.2 and K_V _1.6. But the insensitivity to 5 mM TEA, which should have blocked K_V _1.1, K_V _1.6, K_V _3.1–3.4 and at least partially K_V _2.1 and 2.2 [37], should exclude K_V _1.1 and 1.6. Consequently, it seemed to be likely that the K_V _1.2 channel is a possible candidate for the hypoxia-sensitive K^+ ^current in small DRG neurones.

First, we tested the neurones for hypoxia-sensitive currents because of the lack of knowledge about the expression pattern of the different K_V _channels in a certain neurone. Subsequently, we applied a hypoxic solution containing the specific toxin to those neurones which revealed reduced K^+ ^currents (Table 1). In another series of experiments we applied the toxin first to ensure that the K_V_channels sensitive to the toxin were expressed and functional. After blockade by the toxin, we superfused with a hypoxic solution containing the same toxin (Table 2). We used the following toxins: DTX-k (25 nM), a selective blocker of the cloned K_V _1.1 channel [39], TsTX-K (20 nM) a blocker of K_V _1.2 [40] and 20 nM MgTX as blocker of K_V _1.3 [41] which has been shown to be oxygen-sensitive in lymphozytes and PC12-cells [42,43]. Finally, we tested 10 nM AgTX-2 as blocker of the K_V _1.6 channel which is blocked by 10 nM AgTX-2 [44]. As AgTX-2 has been shown to block the K_V_1.1 and 1.3, too, we added 20 nM MgTX and 25 nM DTX-K to all control solutions to block K_V_1.1 and 1.3 first and to exclude an additional effect of AgTX-2 on these channels. If hypoxia completely blocks a specific channel, subsequent application of another inhibitor (i.e. the blocking toxin) should not have any further effect (experiments shown in table 1). On the other hand, if the K_V _channel is already blocked by its specific channel blocker, hypoxia can not block this channel any more. None of the peptide toxins mentioned in tables 1 and 2 fulfil both criteria. Taken together, the results shown in tables 1 and 2 fail to narrow down the hypoxia- and DTX-sensitive K^+^-channel to one of the genetically and pharmacologically defined homomeric K_V _channels.

**Table 1:** Application of hypoxia and subsequently of toxin.

	**Control**	**Hypoxia**	**Washout**	**Toxin**	**Hypoxia+ Toxin**	**Washout**
**α-DTX (n = 7)**	2.92 ± 0.73	2.36 ± 0.69 ^†^	2.53 ± 0.66 (n.s.)	1.64 ± 0.51 ^†^	1.60 ± 0.48 (n.s.)	n.d.
**TEA (n = 8)**	4.12 ± 0.62	3.65 ± 0.52 ^†^	3.34 ± 0.48 (n.s.)	2.02 ± 0.35 ^†^	1.79 ± 0.32 *	n.d.
**DTX-K (n = 6)**	3.14 ± 0.32	2.46 ± 0.28 ^†^	n.d.	n.d.	2.08 ± 0.31 *	1.56 ± 0.63 (n = 2, n.d.)
**TsTX-K (n = 5)**	2.91 ± 0.38	2.53 ± 0.43 *	n.d.	n.d.	1.92 ± 0.34 ^†^	1.77 ± 0.20 (n.s.)
**MgTX (n = 7)**	2.41 ± 0.48	1.89 ± 0.37 *	n.d.	n.d.	1.55 ± 0.30 (n.s.)	1.82 ± 0.59 (n.s.)
**AgTX-2 (n = 2)**	2.48	2.15	n.d.	n.d.	1.61 ± 0.17	1.25 ±

K^+ ^current amplitude of different neurones in nA ± S.E.M. * p < 0.05, ^† ^p < 0.01 when tested against the preceding value, n.s. = not significant, n.d. not determined

**Table 2:** Application of toxins first and subsequently of hypoxia.

	**Control**	**Toxin**	**Toxin+Hypoxia**	**Washout**
**DTX-K (n = 4)**	2.43 ± 1.49	1.27 ± 0.64	0.94 ± 0.15	1.20 ± 0.20
**TsTX-K (n = 4)**	2.67 ± 0.28	2.25 ± 0.29*	1.80 ± 0.34*	1.49 ± 0.57
**MgTX (n = 5)**	2.91 ± 0.51	2.17 ± 0.46*	1.33 ± 0.48*	1.35 ± 0.66
**AgTX-2 (n = 4)**	2.97 ± 0.33	2.71 ± 0.29*	2.25 ± 0.30^†^	2.06 ± 0.29

Outward current amplitude of different neurones in nA ± S.E.M. * p < 0.05, ^† ^p < 0.01 when tested against the preceding valuecolumn, n.s. = not significant

These experiments demonstrate that hypoxia reduces a voltage-gated K^+ ^current in small DRG neurones which is sensitive to DTX. This current cannot be further assigned to one of the known DTX sensitive K_V _channels.

## Discussion

In contrast to the growing knowledge about the effects of hypoxia on neurones of the CNS there is only little data present about its effects on the PNS. However, there are several clinically important conditions where nerve fibers or neurones of the PNS are exposed to reduced levels of oxygen which are mainly caused by a reduced blood flow due to an obstruction or compression of blood vessels or a direct compression of the neuronal tissue [24,45]. The slice preparation [30,32,46] allows access to a more intact preparation of DRG neurones, especially to those connected to Aδ and C-fibres conducting afferent input like pain, temperature and tactile information (Fig. 1). It is thus possible to describe whole-cell currents with their functional contribution to APs without any enzymatic pretreatment of the neurones.

Our results demonstrate that hypoxia blocks DTX-sensitive K^+ ^currents in small DRG-neurones and describe the functional consequences of this blockade.

### Small DRG neurones respond non-uniformly to moderate hypoxia

We focused in this study on the effect of moderate hypoxia on small DRG neurones because this might be the more common pathophysiological situation. In most situations a moderate hypoxia might precede a later developing severe hypoxia or anoxia which leads to different and more severe responses of neurones as apoptosis and necrosis [47]. During a dramatic reduction of pO_2 _level in the Ringer's solution we found a complex change in those neurones which responded with repetitive firing. Beside a lowering of threshold and an earlier adaptation of firing (Fig. 2) we observed a decrease in outward currents. However, the reduction of the outward current at potentials > -50 mV contributed mainly to changes in the AP shape und increased after potentials, therefore leading to pronounced inactivation of Na^+ ^currents and reduced firing frequency at higher current stimuli. On the other hand it is unlikely that these currents contribute to the changes in threshold.

During application of moderate hypoxia on DRG neurones in physiological external solution they responded within tens of seconds with a change of the outward current. Whereas about one third of the neurones showed a reversible activation of an outward current, the majority of cells displayed a reduction in outward current which was only partially reversible in most neurones during the time course of patch clamp experiments (Fig. 3, 4). This non-uniform response to hypoxia was not surprising because small DRG neurones are known to be inhomogeneous with respect to expression of different types of ion channels and they conduct different types of sensory stimuli (K^+ ^channels [29,32,35,36], Na^+ ^channels [48], Ca^2+ ^channels [49]). The lower oxygen concentration used in this study (21% O_2 _compared to 95% O_2 _in most other studies) did not seem to affect the neurones under control conditions. The mean resting potential of -68.0 ± 1.8 mV was even more negative than that of neurones in a former study [50] under "high oxygen" (-60.9 ± 1.5 mV, n = 11 group A neurones) and the mean AP duration of 3.08 ± 0.31 ms was in between the values of the two subgroups in the former study (3.45 ± 0.34 ms for 11 group A neurones and 2.47 ± 0.16 ms for 20 group B neurones [50]). Taken together this indicates that cell under 21% O_2 _were comparably healthy to cells investigated under carbogen bubbling [32].

### Voltage-dependent K^+ ^channels and hypoxia

The observed changes in whole-cell outward currents could have been either due to changes in inward or outward currents which are overlapping under the experimental conditions. By replacing extracellular Na^+ ^with choline chloride and blockade of Ca^2+ ^currents by intracellular fluoride we could assign the main part of the observed changes in whole cell currents to an activation and an inhibition of K^+ ^currents in the two groups of neurones, respectively (Fig. 5). Only a few neurones did not reveal changes of K^+ ^currents during hypoxia (Figs. 5C and 6).

Whereas a minor group of neurones showed a reversible increase in K^+ ^current, about two-thirds of neurones displayed a hypoxia-induced depression of K^+ ^currents which was mainly irreversible during the time course of our experiments (Fig. 5).

The only partial recovery of the hypoxia-induced decline of K^+ ^currents (Fig 3A, 4A, 7A) is in contrast to former observations of mainly reversible actions of hypoxia on K_V _channels [11,12]. It might be possible that some factors prolong the blockade of potassium channels which might not be present in expression systems. Additionally, in about 25% of experiments either in Ringer's solution or cholinchlorid the neurones showed a reversible increase of currents under hypoxia. We think that the reversibility of these experiments together with the stable currents in the "unchanged" group are clear hints for a specific effect of hypoxia. Furthermore, we cannot exclude that the observed partial recovery is just a slow or delayed reversibility that might have be seen at longer time courses of the washout period [20]. Differences in recovery from ischaemia-induced effects have been reported for sensory and motor axons of human median nerve where the sensory neurones need a longer time period to return to pre-ischaemic excitability [51].

Beside K_V _channels other channels might contribute to the hypoxia induced responses under physiological conditions. Hypoxia-induced increase of cytoplasmatic Ca^2+ ^has been described for carotid body type I cells [52] and particularly for DRG neurones where it has been shown that the hypoxia-induced increase in intracellular Ca^2+ ^concentration is due to an influx through L-type Ca^2+ ^channels [5]. Beside stimulating the production and release of NO by endothelial NO synthase in DRG neurones [25] this might influence Ca^2+^-dependent channels and other Ca^2+^-dependent mechanisms (e.g. kinases). However, changes of internal Ca^2+ ^in those experiments with K_V _channels are unlikely due to the internal fluoride which binds any free internal Ca^2+ ^most effectively [53]. Because the relative reduction in presence of choline chloride was similar to that in Ringer's solution (25% vs. 29%) it supports the idea that the major component is a voltage-dependent K^+ ^current. The hypoxia sensitive current activated mainly at potentials positive to -50 mV (Figs. 3 and 5A) with voltage-dependent characteristic which makes a contribution of one the two-pore-domain K^+ ^channels rather unlikely.

### The hypoxia-sensitive current is DTX sensitive but insensitive to other specific blockers

α-DTX has been described to block K_V _1.1, 1.2 and 1.6 and 5 mM TEA should have blocked K_V _1.1, K_V _1.6, K_V _3.1–3.4 and at least partially K_V _2.1 and 2.2, too [37]. It has been shown previously in ganglia nodosa that the α-DTX-sensitive group of K_V _channels is a subcomponent of the 4-AP sensitive K^+ ^current, therefore we have not used 4-AP [54]. Consequently, it seemed to be likely that the K_V _1.2 channel is responsible or contributes to the hypoxia-sensitive K^+ ^current in small DRG neurones.

Due to the problem of inhomogeneous distribution of different K_V _channels in a specific neurone [35,36], we tried two different sequences of hypoxia and toxin application (tables 1 and 2). The hypoxia-sensitive K_V _channel should neither show a further reduction in toxin after application of hypoxia (table 1) nor in subsequent hypoxia after application of the toxin (table 2). If there was no further change in hypoxia after application of the toxin, the toxin-sensitive current could be the demanded K_V _channel type already blocked by hypoxia (table 1). In case of a further reduction there had to be another K^+ ^channel, which is not blocked by this toxin, contributing to the hypoxia-sensitive K^+ ^current in that specific neurone.

Even though α-DTX could completely prevent the hypoxia effect, none of the other toxins had a similar effect on hypoxia-induced reduction of the K^+ ^current. According to the literature the toxin concentrations used in this study should have been high enough to get a sufficient complete inhibition of the sensitive K_V _channel. Therefore we cannot assign the hypoxia-sensitive K^+ ^current to one single K_V _α-subunit as described in expression systems. This could be due to different K_V _α-subunits which are expressed in the same neurone and have assembled to heteromultimeric channels. Compared to homomultimera these heteromultimeric channels can have altered channel properties and toxin sensitivity. This has been demonstrated for a heteromultimer of K_V_1.2/K_V_1.5 [12] and other heteromultimera of voltage-gated K^+ ^channels where an enhanced sensitivity to dendrotoxin has been demonstrated for the heteromultimer compared to the homomultimer [55-57].

Therefore we suggest that the hypoxia-sensitive and α-DTX sensitive K^+ ^channel in our preparation is the correlate to a heteromultimeric channel assembled from different K_V _channel α-subunits present in DRG neurones. Alternatively it is possible that a co-assembled β-subunit has altered some electrophysiological properties of an expressed K_V _channel α-subunit [58,59] and can even confer oxygen-sensitivity to an expressed Kv α-subunit [60]. However, it is nearly impossible with whole-cell recordings to differentiate between "simple" blockade and alterations in gating of the channels.

### Effects of hypoxia on action potentials

Although it is unlikely, that voltage-gated K^+ ^channels are active around the resting membrane potential of small DRG neurones (-60 mV to -70 mV; [50]), the block of K^+ ^channels which becomes active at potentials close to the threshold of APs might influence the excitability of neurones under hypoxic conditions. It has been demonstrated that DTX-sensitive currents modulate action potentials and regulate excitability in rat visceral sensory neurones [61]. Application of DTX had only little effect on the resting membrane potential in that study but influenced the shape of APs, increased the excitability and the firing frequency of neurones from nodose ganglia. DTX-sensitive K_V _channels have been described as fast-activating and slow-inactivating and activate at membrane potentials slightly positive to the resting membrane and close to the threshold for APs under normoxic conditions [61]. These effects on sensory neurones from nodose ganglia were observed even though the DTX-sensitive current fraction was only about 12 – 25 % of the total K^+ ^current.

In our preparation about 42 % (at 0 mV) of the K^+ ^current was DTX sensitive (Fig. 4), therefore the effect of this component on cell excitability might be even higher. There was no correlation with changes of the resting membrane potential but in some neurones we observed an increase in excitability (Figs. 2 and 4). This is unlikely to be caused by the changes at +40 mV but might be explained by minimal changes of the total outward currents active around the threshold potential (see I/V relation of blocked current in Fig 3C). We could observe a prolongation of action potential duration corresponding to the decrease in outward-current in 11 neurones (Fig. 4).

### Other factors influencing the effects of hypoxia on small DRG neurones

It cannot be excluded that part of the effects of hypoxia in slices are due to secondary effects after a local release of neuropeptides under hypoxia. Other investigators do not use synaptic blockers in their experiments with DRG neurones because these neurones normally do not possess synapses. Further experiments in the presence of synaptic blockers might help to clarify if an indirect effect influences the hypoxic modulation of K^+ ^channels.

On the other hand it is reasonable to assume that the observed effects of hypoxia might have been even bigger when tested at a temperature closer to the physiological level (i.e. 37°C) with a higher metabolic rate and oxygen consumption rate of the tissue. Some ion channels have been described to be directly temperature-modulated (e.g. TRPV1, TRPV2, TRPM8, TRAAK, TREK-1 and TREK-2; [62-64]) and might further influence functional responses to hypoxia. Further experiments at body temperature might help to evaluate the effects in more details.

We used young rats (2–9 d) because the slice-technique of dorsal root ganglia is well established for animals of this age [29,32]. The possible changes in ion channel distribution during development of the PNS and CNS make it preferable to test effects on mature animals. However, older animals cannot be investigated easily in the slice technique due to their more compact connecting tissue.

Our findings in small DRG neurones to moderate hypoxia might account for some of the neurological phenomena as paraesthesia, numbness or pain observed under conditions with a reduced oxygen level in the PNS. In fact, the blockade of a part of voltage-gated K_V _channels in small DRG neurones might be one part of the pathophysiological mechanisms which aggravate the situation after the initial response of the sensory neurone to hypoxia.

## Competing interests

The authors declare herewith that they have no financial as well as non-financial competitions with any other people or organizations.
